# 2-(1,3-Benzothiazol-2-ylsulfanyl)-*N*-(2-methylphenyl)acetamide

**DOI:** 10.1107/S1600536812042109

**Published:** 2012-10-20

**Authors:** Yue Sun, Xiao-Jun Wang, Peng-Wu Zheng

**Affiliations:** a School of Pharmacy, Jiangxi Science and Technology Normal University, Nanchang 330013, People’s Republic of China; b Polytechnic Institute of Jiangxi Science and Technology Normal University, Nanchang 330013, People’s Republic of China

## Abstract

In the title mol­ecule, C_16_H_14_N_2_OS_2_, the benzene ring and the benzo[*d*]thia­zole mean plane form a dihedral angle of 75.5 (1)°. The acetamide group is twisted by 47.7 (1)° from the attached benzene ring. In the crystal, mol­ecules related by translation along the *a* axis are linked into chains through N—H⋯O hydrogen bonds.

## Related literature

For the crystal structures of similar compounds, see: Gao *et al.* (2007 ▶); Zhao *et al.* (2009 ▶). For the medical activity of heterocyclic derivatives containing the acetamide group, see: Fallah-Tafti *et al.* (2011 ▶); Shams *et al.* (2011 ▶)
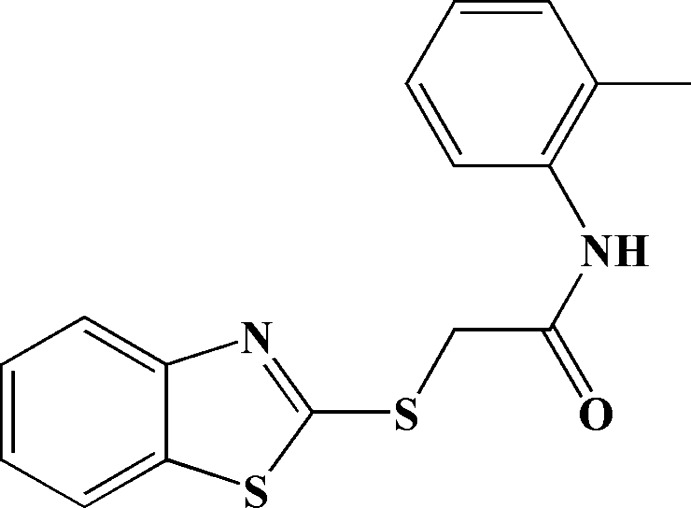


## Experimental

### 

#### Crystal data

C_16_H_14_N_2_OS_2_
*M* *_r_* = 314.41
Monoclinic, 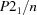

*a* = 4.7957 (8) Å
*b* = 27.496 (4) Å
*c* = 10.9906 (13) Å
β = 97.048 (4)°
*V* = 1438.3 (4) Å^3^
*Z* = 4
Mo *K*α radiation
μ = 0.37 mm^−1^
*T* = 113 K
0.22 × 0.06 × 0.06 mm

#### Data collection

Rigaku Saturn CCD area-detector diffractometer
Absorption correction: multi-scan (*CrystalClear*; Rigaku/MSC, 2005 ▶) *T*_min_ = 0.923, *T*_max_ = 0.978
14718 measured reflections
3421 independent reflections
2923 reflections with *I* > 2σ(*I*)
*R*_int_ = 0.055

#### Refinement

*R*[*F* ^2^ > 2σ(*F* ^2^)] = 0.048
*wR*(*F* ^2^) = 0.100
*S* = 1.06
3421 reflections
195 parameters
H atoms treated by a mixture of independent and constrained refinement
Δρ_max_ = 0.33 e Å^−3^
Δρ_min_ = −0.27 e Å^−3^

### 

Data collection: *CrystalClear* (Rigaku/MSC, 2005 ▶); cell refinement: *CrystalClear*; data reduction: *CrystalClear*; program(s) used to solve structure: *SHELXS97* (Sheldrick, 2008 ▶); program(s) used to refine structure: *SHELXL97* (Sheldrick, 2008 ▶); molecular graphics: *SHELXTL* (Sheldrick, 2008 ▶); software used to prepare material for publication: *SHELXL97*.

## Supplementary Material

Crystal structure: contains datablock(s) global, I. DOI: 10.1107/S1600536812042109/cv5340sup1.cif

Structure factors: contains datablock(s) I. DOI: 10.1107/S1600536812042109/cv5340Isup2.hkl

Supplementary material file. DOI: 10.1107/S1600536812042109/cv5340Isup3.cml

Additional supplementary materials:  crystallographic information; 3D view; checkCIF report

## Figures and Tables

**Table 1:** Hydrogen-bond geometry (Å, °)

*D*—H⋯*A*	*D*—H	H⋯*A*	*D*⋯*A*	*D*—H⋯*A*
N1—H1⋯O1^i^	0.81 (2)	2.10 (2)	2.906 (2)	168 (2)

Symmetry code: (i) 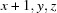.

**Fig. 1.**
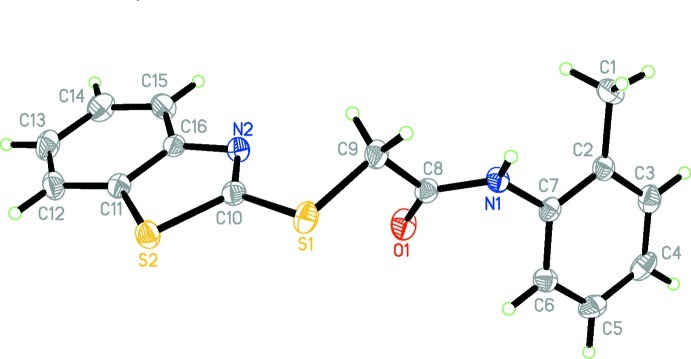
View of the molecule of (I) showing the atom-labelling scheme. Displacement ellipsoids are drawn at the 60% probability level.

## supplementary crystallographic information

### Comment

Acylamide compounds have gained widely attention due to their the important medical activity. Recently, the synthesis and medical activities of some heterocyclic derivatives containing the acylamide moiety have been reported (Fallah-Tafti *et al.*, 2011; Shams *et al.*, 2011). Now the title compound, 2-(benzo[*d*]thiazol-2-ylthio)-N-*o*-tolylacetamide, was synthesized and its crystal structure was reported.

The molecular structure of title compound and the atom-mumbering scheme are shown in Fig. 1. The molecule contain a benzene ring and benzo[*d*]thiazole ring. The dihedral angle between the benzene ring and benzo[*d*]thiazole ring is 75.5°. The acetamide group is twisted at 47.7 (1)° from the attached benzene ring. C1 atom attached to the benzene ring is coplanar to the benzene ring with an r.m.s deviation of 0.0046 Å. As a result of π–π conjugation, the C*_sp_*^2^—S bond [S1—C10 = 1.745 (2) Å] is significantly shorter than the C*_sp_*^3^—S bond [S1—C9 = 1.812 (2) Å]. These values compare with the values of 1.772 (3) and 1.801 (2) Å reported in the literature (Gao *et al.*, 2007; Zhao *et al.*, 2009).

The crystal structure is stablized by the intermolecular N—H···O hydrogen bond (Table 1) interaction.

### Experimental

The title compound was synthesized by the reaction of the benzo[*d*]thiazol-2-thiol with 2-methylphenyl carbamic chloride in the refluxing ethanol. Crystals of (I) suitable for single-crystal X-ray analysis were grown by slow evaporation of a solution in chloroform–ethanol (1:1).

### Refinement

Atom H1 attached to N atom was located on a difference map and refined isotropically. Other H atoms were positioned geometrically (C—H = 0.95–0.99 Å), and refined as riding, with *U*_iso_(H) = 1.2–1.5*U*_eq_(C).

### Crystal data

**Table:**

C_16_H_14_N_2_OS_2_	*F*(000) = 656
*M**_r_* = 314.41	*D*_x_ = 1.452 Mg m^−^^3^
Monoclinic, *P*2_1_/*n*	Mo *K*α radiation, λ = 0.71073 Å
Hall symbol: -P 2yn	Cell parameters from 4445 reflections
*a* = 4.7957 (8) Å	θ = 1.5–27.9°
*b* = 27.496 (4) Å	µ = 0.37 mm^−^^1^
*c* = 10.9906 (13) Å	*T* = 113 K
β = 97.048 (4)°	Prism, colourless
*V* = 1438.3 (4) Å^3^	0.22 × 0.06 × 0.06 mm
*Z* = 4	

### Data collection

**Table:**

Rigaku Saturn CCD area-detector diffractometer	3421 independent reflections
Radiation source: rotating anode	2923 reflections with *I* > 2σ(*I*)
Multilayer monochromator	*R*_int_ = 0.055
Detector resolution: 14.22 pixels mm^-1^	θ_max_ = 27.9°, θ_min_ = 1.5°
φ and ω scans	*h* = −6→6
Absorption correction: multi-scan (*CrystalClear*; Rigaku/MSC, 2005)	*k* = −36→36
*T*_min_ = 0.923, *T*_max_ = 0.978	*l* = −14→14
14718 measured reflections	

### Refinement

**Table:**

Refinement on *F*^2^	Primary atom site location: structure-invariant direct methods
Least-squares matrix: full	Secondary atom site location: difference Fourier map
*R*[*F*^2^ > 2σ(*F*^2^)] = 0.048	Hydrogen site location: inferred from neighbouring sites
*wR*(*F*^2^) = 0.100	H atoms treated by a mixture of independent and constrained refinement
*S* = 1.06	*w* = 1/[σ^2^(*F*_o_^2^) + (0.0351*P*)^2^ + 0.5795*P*] where *P* = (*F*_o_^2^ + 2*F*_c_^2^)/3
3421 reflections	(Δ/σ)_max_ = 0.001
195 parameters	Δρ_max_ = 0.33 e Å^−^^3^
0 restraints	Δρ_min_ = −0.27 e Å^−^^3^

### Special details

**Table:**

Geometry. All e.s.d.'s (except the e.s.d. in the dihedral angle between two l.s. planes) are estimated using the full covariance matrix. The cell e.s.d.'s are taken into account individually in the estimation of e.s.d.'s in distances, angles and torsion angles; correlations between e.s.d.'s in cell parameters are only used when they are defined by crystal symmetry. An approximate (isotropic) treatment of cell e.s.d.'s is used for estimating e.s.d.'s involving l.s. planes.
Refinement. Refinement of *F*^2^ against ALL reflections. The weighted *R*-factor *wR* and goodness of fit *S* are based on *F*^2^, conventional *R*-factors *R* are based on *F*, with *F* set to zero for negative *F*^2^. The threshold expression of *F*^2^ > σ(*F*^2^) is used only for calculating *R*-factors(gt) *etc*. and is not relevant to the choice of reflections for refinement. *R*-factors based on *F*^2^ are statistically about twice as large as those based on *F*, and *R*- factors based on ALL data will be even larger.

### Fractional atomic coordinates and isotropic or equivalent isotropic displacement parameters (Å^2^)

**Table:**

	*x*	*y*	*z*	*U*_iso_*/*U*_eq_	
S1	0.48672 (11)	0.112141 (18)	0.29125 (5)	0.02069 (14)	
S2	0.16105 (11)	0.158557 (18)	0.47479 (5)	0.01966 (14)	
O1	0.1342 (3)	0.09883 (5)	0.03414 (14)	0.0233 (3)	
N1	0.5406 (4)	0.08369 (6)	−0.04653 (15)	0.0162 (4)	
N2	0.1583 (3)	0.19259 (6)	0.25292 (15)	0.0168 (3)	
C1	0.6969 (4)	0.10401 (7)	−0.2868 (2)	0.0224 (5)	
H1A	0.7119	0.1064	−0.3747	0.034*	
H1B	0.8819	0.0965	−0.2424	0.034*	
H1C	0.6295	0.1350	−0.2573	0.034*	
C2	0.4933 (4)	0.06418 (7)	−0.26509 (18)	0.0170 (4)	
C3	0.3715 (4)	0.03516 (7)	−0.36122 (19)	0.0212 (4)	
H3	0.4183	0.0407	−0.4416	0.025*	
C4	0.1835 (4)	−0.00162 (7)	−0.3421 (2)	0.0230 (5)	
H4	0.1010	−0.0206	−0.4092	0.028*	
C5	0.1159 (4)	−0.01059 (7)	−0.2249 (2)	0.0216 (4)	
H5	−0.0119	−0.0359	−0.2115	0.026*	
C6	0.2356 (4)	0.01747 (7)	−0.12783 (19)	0.0181 (4)	
H6	0.1916	0.0112	−0.0473	0.022*	
C7	0.4210 (4)	0.05501 (7)	−0.14794 (18)	0.0157 (4)	
C8	0.3912 (4)	0.10278 (7)	0.03867 (18)	0.0174 (4)	
C9	0.5622 (4)	0.13057 (7)	0.14044 (18)	0.0203 (4)	
H9A	0.5228	0.1658	0.1296	0.024*	
H9B	0.7645	0.1254	0.1346	0.024*	
C10	0.2600 (4)	0.15837 (7)	0.32579 (18)	0.0171 (4)	
C11	−0.0382 (4)	0.21033 (7)	0.43540 (18)	0.0172 (4)	
C12	−0.2001 (4)	0.23768 (8)	0.50696 (19)	0.0221 (4)	
H12	−0.2147	0.2290	0.5896	0.026*	
C13	−0.3391 (4)	0.27784 (8)	0.4536 (2)	0.0243 (5)	
H13	−0.4518	0.2970	0.5004	0.029*	
C14	−0.3167 (4)	0.29069 (8)	0.3323 (2)	0.0243 (5)	
H14	−0.4140	0.3185	0.2980	0.029*	
C15	−0.1551 (4)	0.26352 (7)	0.26125 (19)	0.0217 (4)	
H15	−0.1412	0.2724	0.1786	0.026*	
C16	−0.0131 (4)	0.22287 (7)	0.31355 (18)	0.0157 (4)	
H1	0.710 (5)	0.0869 (8)	−0.034 (2)	0.019 (6)*	

### Atomic displacement parameters (Å^2^)

**Table:**

	*U*^11^	*U*^22^	*U*^33^	*U*^12^	*U*^13^	*U*^23^
S1	0.0214 (3)	0.0197 (3)	0.0205 (3)	0.0055 (2)	0.0010 (2)	−0.0037 (2)
S2	0.0230 (3)	0.0202 (3)	0.0161 (3)	0.0031 (2)	0.0038 (2)	0.00153 (19)
O1	0.0109 (7)	0.0304 (8)	0.0289 (9)	−0.0010 (6)	0.0032 (6)	−0.0092 (7)
N1	0.0101 (8)	0.0202 (9)	0.0186 (9)	−0.0016 (7)	0.0025 (7)	−0.0035 (7)
N2	0.0168 (8)	0.0176 (8)	0.0163 (8)	0.0011 (6)	0.0029 (7)	−0.0007 (7)
C1	0.0207 (11)	0.0236 (11)	0.0238 (11)	−0.0007 (8)	0.0068 (9)	0.0036 (9)
C2	0.0151 (10)	0.0168 (9)	0.0193 (10)	0.0035 (7)	0.0032 (8)	0.0004 (8)
C3	0.0252 (11)	0.0211 (10)	0.0175 (10)	0.0063 (8)	0.0026 (9)	0.0001 (8)
C4	0.0268 (11)	0.0177 (10)	0.0223 (11)	0.0021 (8)	−0.0059 (9)	−0.0055 (8)
C5	0.0179 (10)	0.0170 (10)	0.0292 (12)	−0.0022 (8)	0.0003 (9)	−0.0004 (9)
C6	0.0156 (10)	0.0184 (10)	0.0205 (10)	0.0009 (8)	0.0032 (8)	0.0015 (8)
C7	0.0129 (9)	0.0148 (9)	0.0191 (10)	0.0031 (7)	0.0003 (8)	−0.0021 (8)
C8	0.0156 (10)	0.0167 (9)	0.0199 (10)	0.0000 (8)	0.0027 (8)	−0.0022 (8)
C9	0.0139 (10)	0.0233 (10)	0.0243 (11)	−0.0013 (8)	0.0051 (9)	−0.0074 (8)
C10	0.0151 (9)	0.0182 (9)	0.0178 (10)	−0.0023 (8)	0.0019 (8)	−0.0027 (8)
C11	0.0158 (10)	0.0177 (9)	0.0179 (10)	0.0000 (8)	0.0017 (8)	−0.0015 (8)
C12	0.0214 (11)	0.0269 (11)	0.0189 (10)	0.0001 (9)	0.0070 (9)	−0.0041 (9)
C13	0.0196 (11)	0.0253 (11)	0.0284 (12)	0.0023 (9)	0.0050 (9)	−0.0088 (9)
C14	0.0229 (11)	0.0201 (10)	0.0294 (12)	0.0062 (8)	0.0010 (9)	−0.0004 (9)
C15	0.0226 (11)	0.0217 (10)	0.0215 (11)	0.0025 (8)	0.0056 (9)	0.0026 (8)
C16	0.0137 (9)	0.0159 (9)	0.0179 (10)	−0.0013 (7)	0.0027 (8)	−0.0021 (7)

### Geometric parameters (Å, º)

**Table:**

S1—C10	1.745 (2)	C4—H4	0.9500
S1—C9	1.812 (2)	C5—C6	1.383 (3)
S2—C11	1.739 (2)	C5—H5	0.9500
S2—C10	1.760 (2)	C6—C7	1.398 (3)
O1—C8	1.232 (2)	C6—H6	0.9500
N1—C8	1.353 (2)	C8—C9	1.511 (3)
N1—C7	1.427 (2)	C9—H9A	0.9900
N1—H1	0.81 (2)	C9—H9B	0.9900
N2—C10	1.292 (2)	C11—C12	1.392 (3)
N2—C16	1.395 (2)	C11—C16	1.402 (3)
C1—C2	1.505 (3)	C12—C13	1.382 (3)
C1—H1A	0.9800	C12—H12	0.9500
C1—H1B	0.9800	C13—C14	1.396 (3)
C1—H1C	0.9800	C13—H13	0.9500
C2—C3	1.394 (3)	C14—C15	1.385 (3)
C2—C7	1.397 (3)	C14—H14	0.9500
C3—C4	1.388 (3)	C15—C16	1.395 (3)
C3—H3	0.9500	C15—H15	0.9500
C4—C5	1.388 (3)		
			
C10—S1—C9	101.26 (9)	O1—C8—N1	123.37 (18)
C11—S2—C10	88.47 (9)	O1—C8—C9	121.60 (17)
C8—N1—C7	123.97 (16)	N1—C8—C9	115.02 (16)
C8—N1—H1	116.7 (16)	C8—C9—S1	112.61 (14)
C7—N1—H1	119.1 (16)	C8—C9—H9A	109.1
C10—N2—C16	109.77 (16)	S1—C9—H9A	109.1
C2—C1—H1A	109.5	C8—C9—H9B	109.1
C2—C1—H1B	109.5	S1—C9—H9B	109.1
H1A—C1—H1B	109.5	H9A—C9—H9B	107.8
C2—C1—H1C	109.5	N2—C10—S1	126.43 (15)
H1A—C1—H1C	109.5	N2—C10—S2	116.76 (14)
H1B—C1—H1C	109.5	S1—C10—S2	116.81 (11)
C3—C2—C7	117.84 (18)	C12—C11—C16	121.74 (18)
C3—C2—C1	121.07 (18)	C12—C11—S2	128.98 (16)
C7—C2—C1	121.10 (18)	C16—C11—S2	109.27 (14)
C4—C3—C2	121.46 (19)	C13—C12—C11	117.73 (19)
C4—C3—H3	119.3	C13—C12—H12	121.1
C2—C3—H3	119.3	C11—C12—H12	121.1
C3—C4—C5	119.99 (19)	C12—C13—C14	121.17 (19)
C3—C4—H4	120.0	C12—C13—H13	119.4
C5—C4—H4	120.0	C14—C13—H13	119.4
C6—C5—C4	119.65 (19)	C15—C14—C13	121.1 (2)
C6—C5—H5	120.2	C15—C14—H14	119.5
C4—C5—H5	120.2	C13—C14—H14	119.5
C5—C6—C7	120.11 (19)	C14—C15—C16	118.57 (19)
C5—C6—H6	119.9	C14—C15—H15	120.7
C7—C6—H6	119.9	C16—C15—H15	120.7
C2—C7—C6	120.93 (18)	N2—C16—C15	124.56 (18)
C2—C7—N1	119.92 (17)	N2—C16—C11	115.72 (17)
C6—C7—N1	119.15 (17)	C15—C16—C11	119.72 (17)
			
C7—C2—C3—C4	0.2 (3)	C9—S1—C10—N2	6.5 (2)
C1—C2—C3—C4	−179.95 (18)	C9—S1—C10—S2	−173.28 (11)
C2—C3—C4—C5	−0.9 (3)	C11—S2—C10—N2	−0.15 (16)
C3—C4—C5—C6	0.4 (3)	C11—S2—C10—S1	179.60 (12)
C4—C5—C6—C7	0.7 (3)	C10—S2—C11—C12	−179.2 (2)
C3—C2—C7—C6	0.9 (3)	C10—S2—C11—C16	0.11 (15)
C1—C2—C7—C6	−178.92 (17)	C16—C11—C12—C13	0.5 (3)
C3—C2—C7—N1	179.88 (17)	S2—C11—C12—C13	179.73 (16)
C1—C2—C7—N1	0.1 (3)	C11—C12—C13—C14	−0.3 (3)
C5—C6—C7—C2	−1.4 (3)	C12—C13—C14—C15	0.2 (3)
C5—C6—C7—N1	179.61 (17)	C13—C14—C15—C16	−0.2 (3)
C8—N1—C7—C2	134.3 (2)	C10—N2—C16—C15	179.80 (19)
C8—N1—C7—C6	−46.7 (3)	C10—N2—C16—C11	−0.1 (2)
C7—N1—C8—O1	−3.0 (3)	C14—C15—C16—N2	−179.45 (18)
C7—N1—C8—C9	178.09 (17)	C14—C15—C16—C11	0.4 (3)
O1—C8—C9—S1	50.5 (2)	C12—C11—C16—N2	179.32 (17)
N1—C8—C9—S1	−130.60 (16)	S2—C11—C16—N2	−0.1 (2)
C10—S1—C9—C8	−100.06 (15)	C12—C11—C16—C15	−0.5 (3)
C16—N2—C10—S1	−179.59 (14)	S2—C11—C16—C15	−179.92 (15)
C16—N2—C10—S2	0.1 (2)		

### Hydrogen-bond geometry (Å, º)

**Table:**

*D*—H···*A*	*D*—H	H···*A*	*D*···*A*	*D*—H···*A*
N1—H1···O1^i^	0.81 (2)	2.10 (2)	2.906 (2)	168 (2)

Symmetry code: (i) *x*+1, *y*, *z*.
